# Improved carrier injection of AlGaN-based deep ultraviolet light emitting diodes with graded superlattice electron blocking layers

**DOI:** 10.1039/c8ra06982d

**Published:** 2018-10-16

**Authors:** Byeongchan So, Jinwan Kim, Taemyung Kwak, Taeyoung Kim, Joohyoung Lee, Uiho Choi, Okhyun Nam

**Affiliations:** Convergence Center for Advanced Nano Semiconductor (CANS), Department of Nano-optical Engineering, Korea Polytechnic University 237 Sangidaehak-ro Siheung-si Gyeonggi-do South Korea ohnam@kpu.ac.kr +82 31 8041 0917 +82 31 8041 0710

## Abstract

A DUV-LED with a graded superlattice electron blocking layer (GSL-EBL) is demonstrated to show improved carrier injection into the multi-quantum well region. The structures of modified EBLs are designed *via* simulation. The simulation results show the carrier behavior mechanism of DUV-LEDs with a single EBL (S-EBL), graded EBL (G-EBL), and GSL-EBL. The variation in the energy band diagram around the EBL region indicates that the introduction of GSL-EBL is very effective in enhancing carrier injection. Besides, all DUV-LEDs emitting at 280 nm are grown in the high temperature metal organic chemical deposition system. It is confirmed that the optical power of the DUV-LED with the GSL-EBL is significantly higher than that of the DUV-LED with the S-EBL and G-EBL.

## Abstract 2

DUV-LEDs with a single EBL, graded EBL, and graded superlattice EBL were demonstrated using the high-temperature metal organic chemical vapor deposition system. A DUV-LED with a GSL-EBL showed improved carrier injection into the multi-quantum well region.

## Graphical abstract

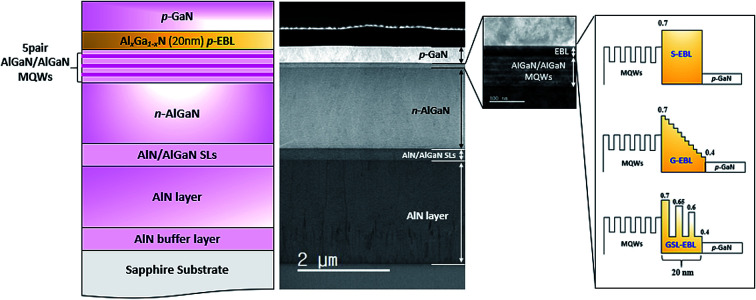

## Introduction

The wavelength of deep ultraviolet (DUV) light (200–280 nm) is very effective in killing germs.^1^ DUV light emitting diodes (DUV-LEDs) have been extensively studied to replace conventional UV sources because the DUV-LEDs have many advantages, such as compact size, fast power toggling, non-toxic material composition, long-lifetime, and low power consumption.^2–4^ However, there are still a few issues that need to be resolved for achieving a high efficiency DUV-LED. The typical external quantum efficiency (EQE) of DUV-LEDs is much lower than that of InGaN-based blue LEDs. Although Takano *et al.* recently investigated the 20% EQE of DUV-LEDs by applying a Rh mirror electrode,^5^ there is scope for improving the efficiency of DUV-LEDs. The internal quantum efficiency (IQE), current injection efficiency (CIE), and light extraction efficiency (LEE) are essential for realizing high EQE DUV-LEDs. Furthermore, many groups have realized improvements in CIE for DUV-LEDs by enhancing doping properties, varying multi-quantum well (MQW) structures, and controlling the electron blocking layer (EBL) growth conditions.^6–10^

As a method for enhancing the CIE of DUV-LEDs, modification of the EBL structure is a very promising solution.^11–13^ Hirayama *et al.* demonstrated high efficiency DUV-LEDs using the multi-quantum barrier EBL, showing the EBL is critical factor to increasing the CIE of DUV-LEDs.^14^ Some groups have also studied the graded superlattice EBL (GSL-EBL) for use in high-performance InGaN LEDs.^15,16^ Park *et al.* reported that the GSL-EBL is needed for the smoother transport of hole compared to SL-EBL.^17^ B. Janjua *et al.* reported that the GSL-EBL is effective to enhance carrier injection for UVC-LED through simulation study.^18^ However, the DUV-LED structure growth with GSL-EBL has not been reported. In this study, we suggested AlGaN based DUV-LED grown with GSL-EBL for enhancing current injection and compared the experimental data with the simulation results. The simulation results show that smooth hole injection and effective blocking of electrons was achieved by insertion of the GSL-EBL. In addition, DUV-LEDs with a single EBL (S-EBL), graded EBL (G-EBL), and GSL-EBL were demonstrated using the high-temperature metal organic chemical vapor deposition (HT-MOCVD) system.

## Experimental section

**Fig. 1:**
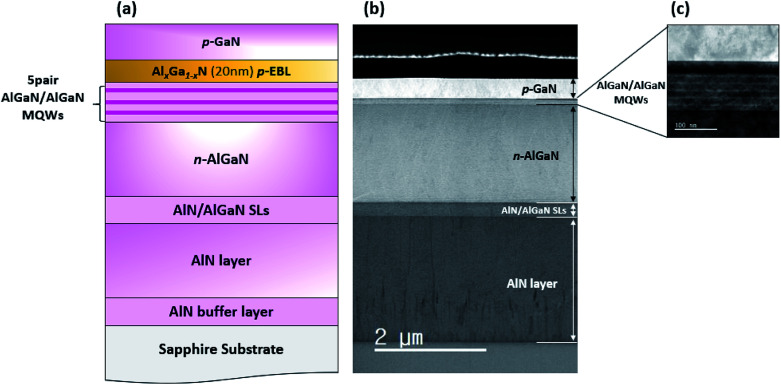
(a) Schematic image of the DUV-LED and cross-section TEM image for (b) the DUV-LED structure and (c) the AlGaN/AlGaN MQWs, respectively.

**Fig. 2:**
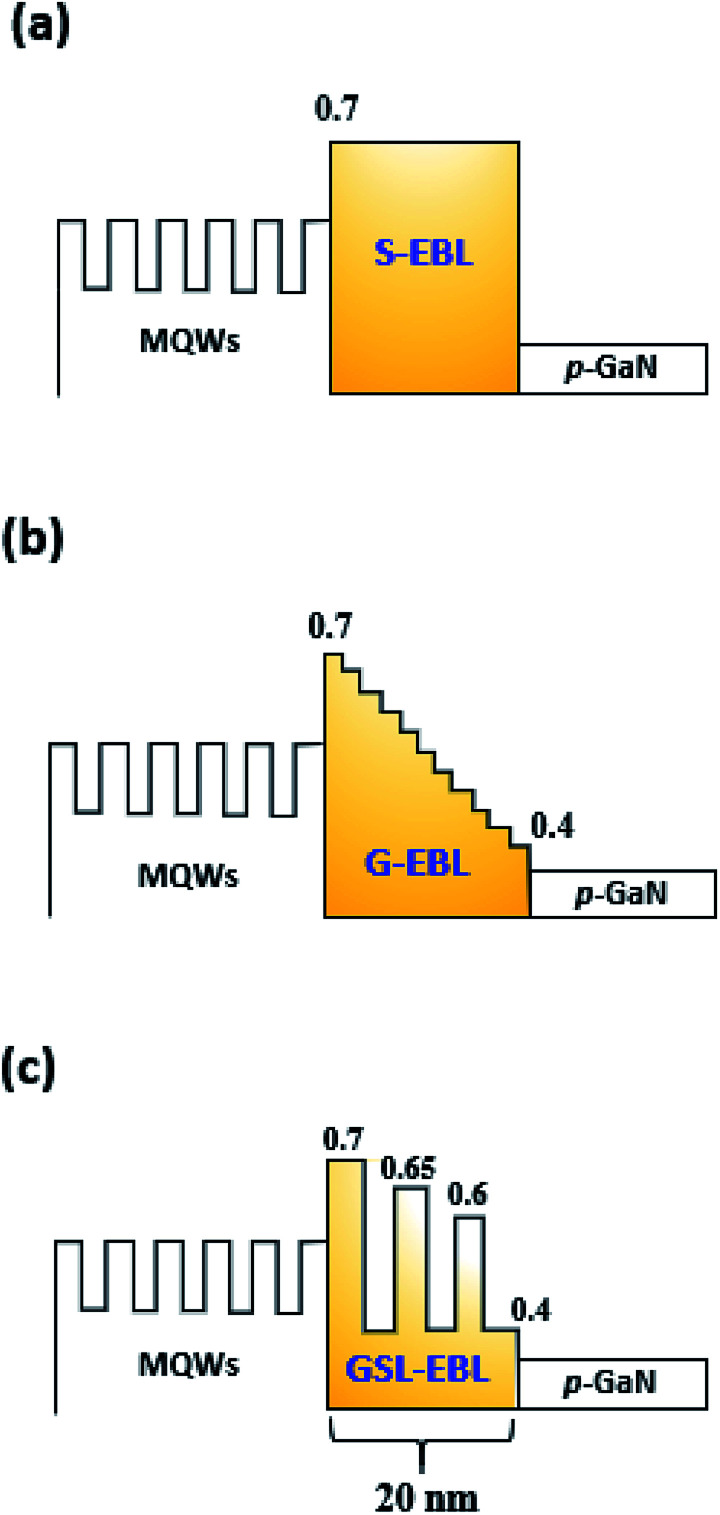
Schematic diagrams of the DUV-LED with (a) S-EBL, (b) G-EBL and (c) GSL-EBL.

In this work, we have grown AlGaN based DUV-LED epi structures as shown in Fig. 1(a) using the HT-MOCVD (Top Engineering, PHAETHON 100U) reactor. On-axis *c*-plane sapphire was used for epitaxy of the DUV-LED structure. Trimethylaluminum (TMAl), trimethylgallium (TMGa), and ammonia (NH_3_) were used to grow AlGaN based materials as the precursors. The p-type and n-type dopant sources were bis-cyclopentadienyl magnesium (CP_2_Mg) and silane (SiH_4_), respectively. H_2_ thermal cleaning was performed on the sapphire substrate in the MOCVD reactor before the growth step. A low-temperature (LT) AlN buffer layer with 25 nm was grown on thermally cleaned sapphire. After that, a 2.2 μm AlN epilayer was grown at 1250 °C. The full width at half maximums (FWHMs) of the X-ray rocking curve (XRC) for the AlN layer were 110 and 460 arcsec for (002) and (102), respectively. Then, 60-pair superlattice layers (SLs) made up of Al_0.7_Ga_0.3_N (1.5 nm)/AlN (4 nm) were grown on an AlN layer to control the strains and reduce dislocations of the n-type AlGaN cladding layer. A 1.8 μm Si-doped *n*-Al_0.56_Ga_0.44_N layer was grown on the SLs. Afterwards, five-period MQWs consisting of 2 nm Al_0.47_Ga_0.53_N wells and 12 nm Al_0.56_Ga_0.44_N barriers were grown on the *n*-AlGaN layer. Then, the S-, G-, and GSL-EBLs with Mg doping were grown after the growth of MQWs. The S-EBL consisted of a 20 nm Al_0.7_Ga_0.3_N layer, and the G-EBL has 12 steps from Al 70% to 40%. The GSL-EBL is formed with AlGaN/AlGaN SLs including three identical wells (Al 40%) and three different barriers (Al 70, 65, 60%). Fig. 2 shows the schematic images of DUV-LEDs with S-, G-, and GSL-EBL. Finally, the *p*-GaN layer was deposited as the hole supplier layer. Fig. 1(b) shows the transmission electron microscope (TEM) image of the DUV-LED structure. The entire layer and interface were clearly observed. In addition, the enlarged TEM images of the 5-pair AlGaN/AlGaN MQWs is shown in Fig. 1(c). Following the growth of *p*-GaN, an *in situ* annealing step carried out in N_2_ ambient at 850 °C for all samples.

The SiLENSe 5.2.1 software was used to verify the carrier transport mechanism in the DUV-LED structure, which was grown by our HT-MOCVD system. A one-dimensional drift-diffusion model of carrier transport is assumed in the LED operation.^19^ The equations between the carrier fluxes and the gradients of the quasi-Fermi level are represented as follows:^20^
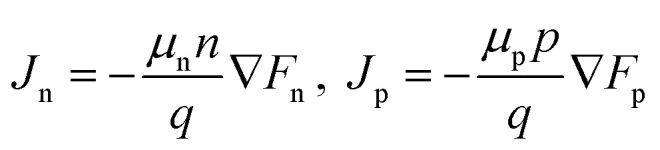


Here, *J*_n_ and *J*_p_ are the electron and hole fluxes, *μ*_n_ and *μ*_p_ are the electron and hole mobilities, *n* and *p* are the electron and hole concentration, and *F*_n_ and *F*_p_ are the electron and hole quasi-Fermi levels. The concentrations of donors and acceptors were 5 × 10^18^ and 2 × 10^19^ cm^−3^, respectively, in the simulation. The ratio of the band offset was 7/3. We applied a constant mobility of 100 cm^2^ V^−1^ s^−1^ for electrons and 5 cm^2^ V^−1^ s^−1^ for holes. The non-radiative carrier lifetime was set as 5 ns, which strongly depends on the epi-layer quality. Other parameters were determined by program defaults.^21^

## Results and discussion

**Fig. 3:**
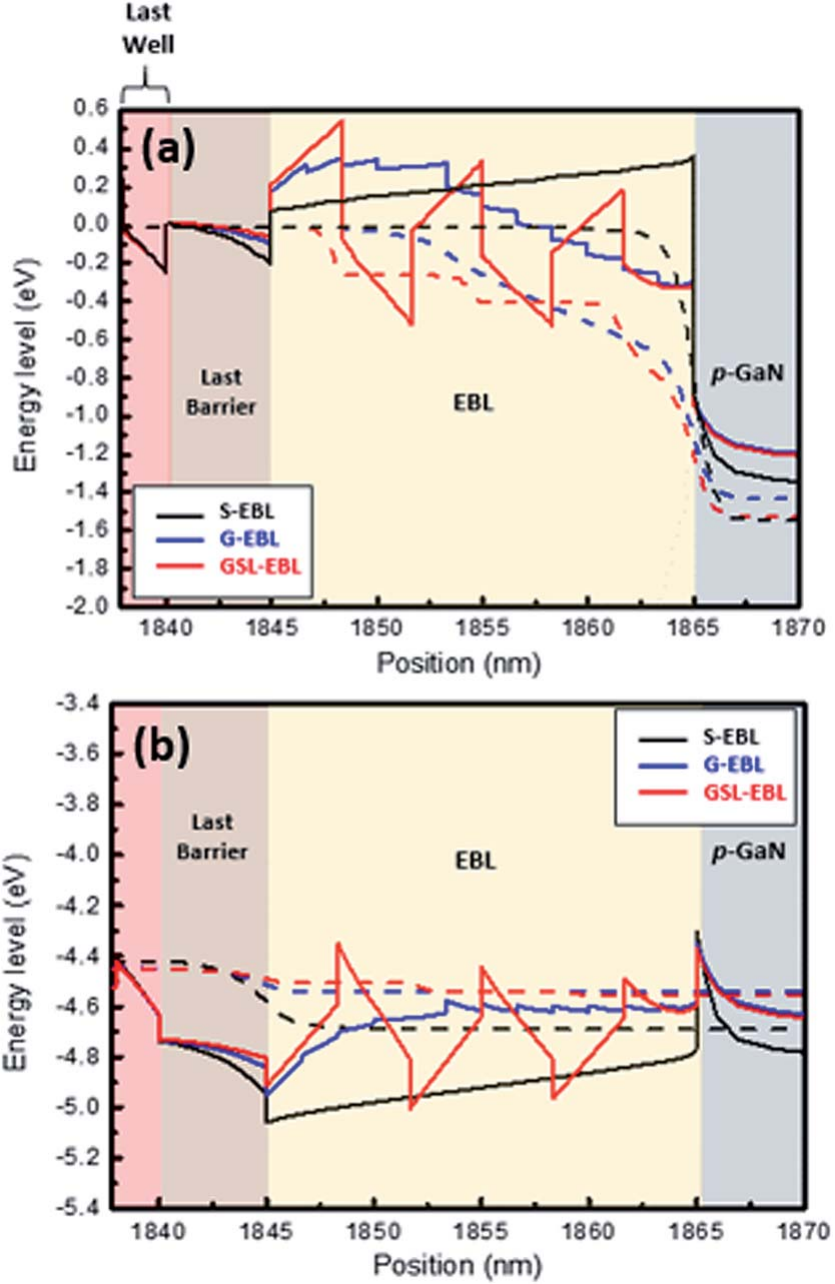
Energy band diagrams of (a) conduction band and (b) valence band around the EBL region for all DUV-LEDs. The solid line and dashed line indicate the conduction (valence) band level and quasi-Fermi energy level, respectively, at 55.5 A cm^−2^.

A simulated energy band diagrams around EBL for DUV-LED with S-EBL, G-EBL, and GSL-EBL are shown in Fig. 3. Fig. 3(a) shows the quasi-Fermi level of the conduction band and the energy band level of electrons for the DUV-LED with S-, G-, and GSL-EBLs, respectively. The potential barrier height for the electron (hole) is defined as the difference between the conduction (valence) band level and quasi-Fermi energy level.^22^ Of all LEDs tested, the GSL-EBL shows the highest potential electron barrier height (≈555 meV), which indicates that effective electron blocking was enabled by the GSL-EBL. In addition, the LED with GSL-EBL shows the smallest parasitic electron reservoir volume at the interface between the last barrier and EBL, which is formed by the polarization effect.^23^ Although the potential barrier of the LED with G-EBL (≈356 meV) is lower than that of the LED with S-EBL (≈377 meV), the volume of the parasitic electron reservoir of the LED with the G-EBL is smaller than that of the LED with the S-EBL. Additional reduction of the electron overflow is proceeded by the suppression of the parasitic electron reservoir.^24^ These simulation results show that the introduction of the GSL-EBL is effective in blocking electron overflow and controlling the electron reservoir volume. Fig. 3(b) shows the quasi-Fermi level of the valence band as well as the energy band level of holes for the DUV-LEDs with the S-, G-, and GSL-EBL, respectively.

**Fig. 4:**
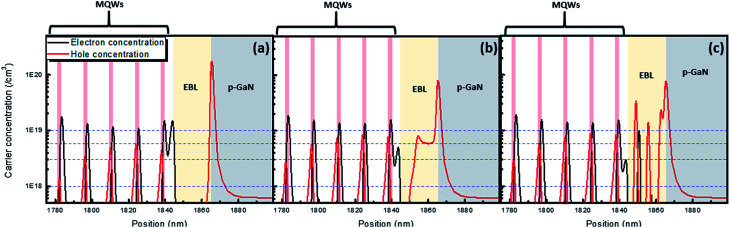
Calculated carrier concentration for DUV-LEDs with (a) S-EBL, (b) G-EBL and (c) GSL-EBL at 55.5 A cm^−2^. The pink lines indicate the active regions in the MQWs.

Although there are notches at the interface between *p*-GaN and the EBL in all samples, only the DUV-LED with the GSL-EBL has three additional notches exceeding the quasi-Fermi energy level of the valence band. It is assumed that the holes are generated in the region of the valence band level exceeding quasi-Fermi energy level.^25^ Therefore, the number of notches surpassing the quasi-Fermi energy level indicates that hole accumulation is favorable. Additionally, GSL-EBL has the lowest spike height (≈423 meV) at the interface between the EBL and last barrier in all the samples. The low spike height implies easier hole injection from the *p*-GaN/EBL to the MQWs. The G-EBL does not have the notch in the EBL, but it has a lower spike height (≈438 meV) than the S-EBL (≈481 meV). Consequently, the simulated energy band diagram indicates that the GSL-EBL is superior in blocking electrons from the MQWs and injecting holes from *p*-GaN to other EBLs. We observed the concentration of holes and electrons in the region of the EBL and MQWs to understand the effect of the modified EBLs (Fig. 4).

The hole carrier concentration of the DUV-LED with the S-EBL at the interface between *p*-GaN and the EBL is highest among all samples. However, this concentration decreases rapidly in the EBL region, as shown in Fig. 4(a). On the other hand, the hole concentration in the G-EBL is reduced to about one-tenth in the middle of G-EBL. Finally, the hole concentration gradually decreases closer to the MQWs. Interestingly, the hole concentration of the DUV-LED with the GSL-EBL increases as closer to the MQWs. This phenomenon can be explained by the energy band diagram. The points at which the hole concentration rapidly increased are in accordance with the notches over the quasi-Fermi energy level of the valence band, and high hole concentration in the GSL-EBL causes a high injection rate of holes in the MQWs region.

**Fig. 5:**
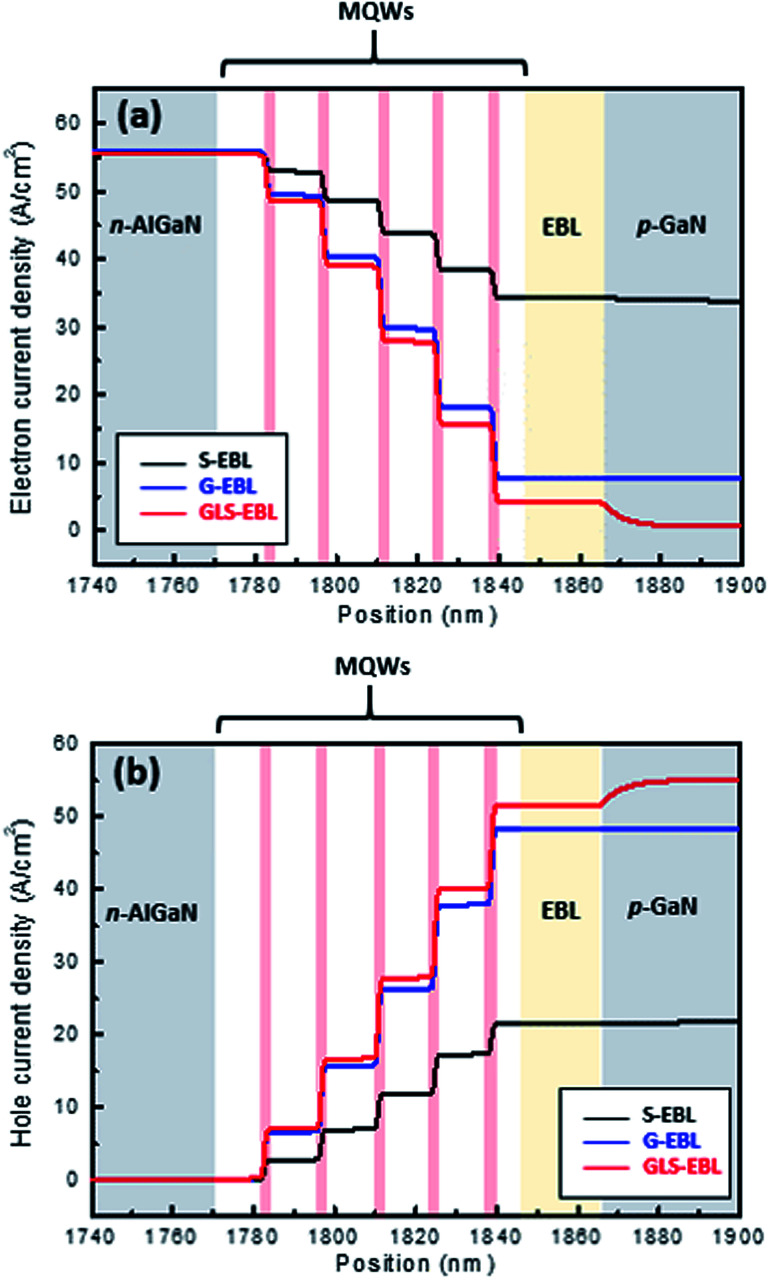
(a) Electron current density and (b) hole current density of DUV-LED with S-EBL, G-EBL, and GSL-EBL at 55.5 A cm^−2^.

Fig. 5 shows the electron and hole current densities, respectively. The electron current densities at the MQWs in GSL-EBL sample is the lowest because of the highest recombination with holes due to the better hole injection through the GSL-EBL layer. The electron current of the DUV-LED with the GSL-EBL is drastically declined, which means that hole injection in the GSL-EBL is more likely than hole injection in the other samples. Moreover, the electron current density in the EBL region is very low in the DUV-LED with the GSL-EBL. These excess electron currents without recombination in the MQWs indicate the amount of electron overflow. The hole current density varies with the type of EBL. The DUV-LED with the GSL-EBL shows the highest hole current injection in all samples, as shown in Fig. 5(b), which is in agreement with the results of Fig. 3(b) and 4(c). These results represent the enhanced recombination of holes and electrons in MQWs.

**Fig. 6:**
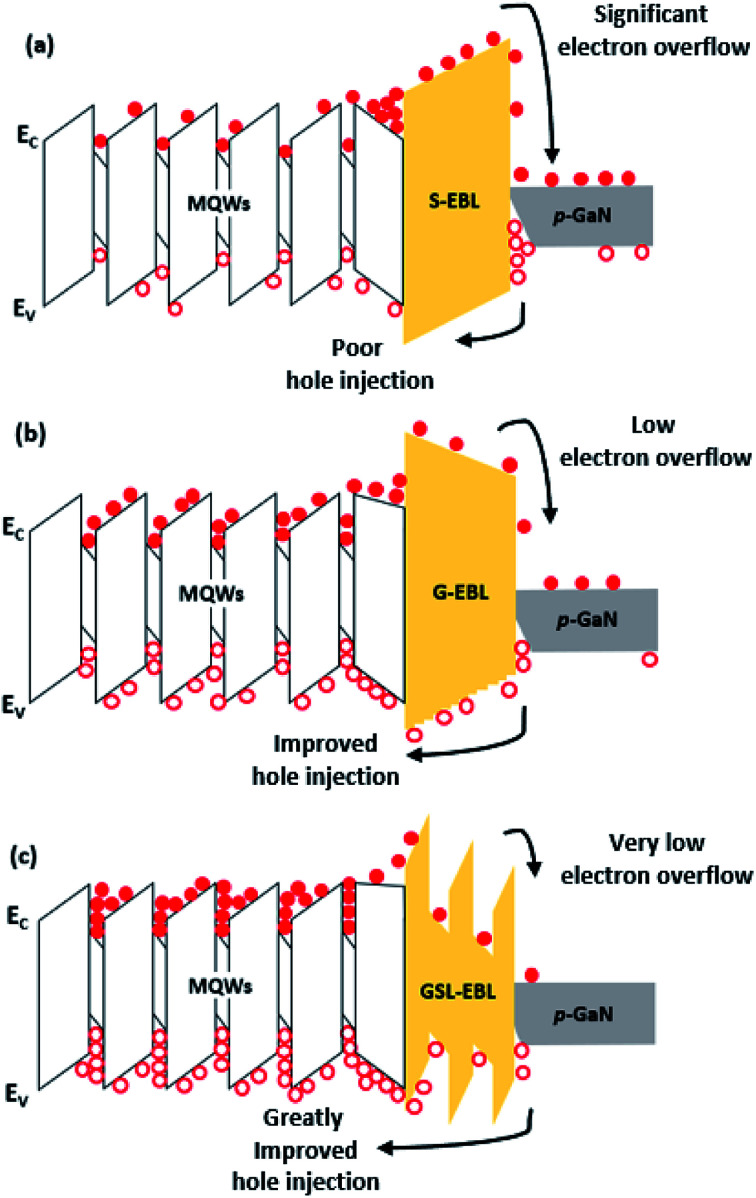
Schematic diagram of the carrier dynamic phenomena of DUV-LED with (a) S-EBL, (b) G-EBL, and (c) GSL-EBL, respectively.

Fig. 6 shows the schematic images of DUV-LEDs and describes the different carrier transport mechanisms by applying different EBL structures in the band of the diagram. The structures of the DUV-LEDs are identical, except for the slightly modified energy band due to the different EBLs. The DUV-LED with the S-EBL has a large parasitic electron reservoir between the last barrier and the EBL due to energy band bending. Thus, many electrons are trapped in the reservoir. Additionally, the low energy level height of the S-EBL induces electron overflow. Meanwhile, the spike height in the valence band makes it difficult for the holes from *p*-GaN to pass the S-EBL. Fig. 6(b) indicates the carrier transport behavior in the DUV-LED with the G-EBL. In the conduction band, the height of the G-EBL energy level is similar to that of the S-EBL. However, the electron concentration that is able to reunite with holes in the MQWs region is increased because the parasitic electron reservoir is considerably reduced in the DUV-LED with G-EBL, and the low height of the spike between the last barrier and the G-EBL in the valence band helps enhance hole injection rate. The GSL-EBL has the highest energy level among all EBLs, so the electron overflow is remarkably reduced. In addition, the effect of the parasitic electron reservoir was not significant, which led to an improvement of the electron concentration in the MQWs region. The hole concentration is increased further in the MQWs region of the DUV-LED with the GSL-EBL, as shown in Fig. 6(c).

**Fig. 7:**
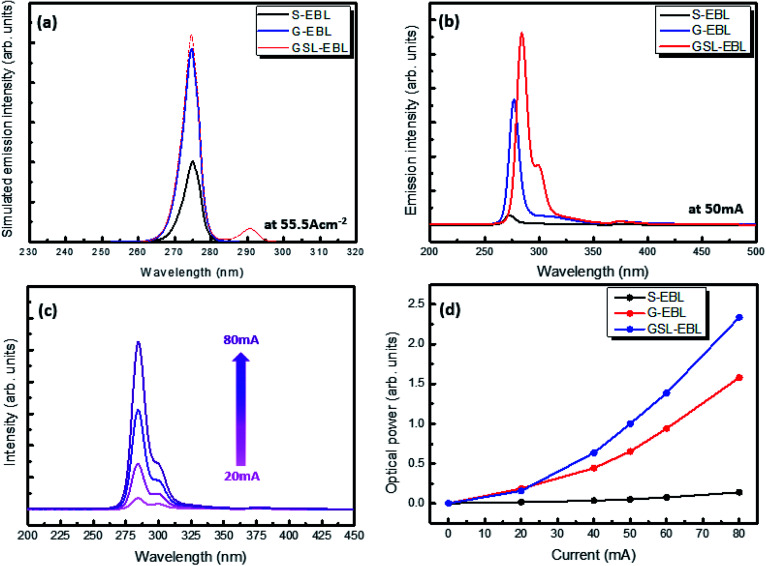
(a) Simulated and (b) experimental emission spectra of DUV-LEDs with S, G, and GSL-EBLs. (c) Emission intensities of GSL-EBLs depending on the current increase. (d) Optical power of DUV-LED with S-, G-, and GSL-EBLs.

There appear to be two causes for the increase in hole concentration: (1) the polarization effect in the band diagram due to the difference in Al composition between the well and the barrier of the GSL-EBL, which also make relatively low activation energy of Mg in the well region,^18,26^ (2) tunneling effect of holes caused by the relatively thin barrier thickness of the GSL-EBL.^27^ The simulated injection efficiencies of the DUV-LED structures with the S-EBL, G-EBL, and GSL-EBL are approximately 37%, 82%, and 88%, respectively. These results indicate that the injection efficiency when the GSL-EBL is used is two times higher than that when the S-EBL is used. Fig. 7 shows the electroluminescence (EL) data of DUV-LEDs grown by HT-MOCVD. We fabricated an on-wafer test structure using indium as contact on the n-type and p-type cladding layers. The optical fiber is located at the bottom of the sample to measure the light emitting downward. The simulated EL spectra of DUV-LEDs with the S-EBL, G-EBL, and GSL-EBL are shown in Fig. 7(a). The emission intensity of the DUV-LED with the GSL-EBL is higher than that of the DUV-LEDs with the S-EBL and G-EBL. It is also shown the parasitic emission peak (≈292 nm) in the DUV-LED with the GSL-EBL, which is attributed to carrier recombination in the well of the GSL-EBL. Fig. 7(b) shows the experimental EL spectra. The experimental results show the different peak wavelengths depending on the types of EBL. The peak wavelength of DUV-LED with GSL-EBL is red-shifted approximately 5 nm from other samples. The reason is considered that the GSL-EBL relaxed compressive strain of MQWs, which was confirmed by the 0th order peak shift of AlGaN MQWs in the XRD 2theta-omega analysis (data not shown). On the whole, the tendency of the emission intensities for the three samples follows the simulation data. The EL intensity of the DUV-LED with the GSL-EBL is significantly higher than those of the S-EBL and G-EBL. These spectral results indicate that the insertion of the GSL-EBL improves the emission intensity of the DUV-LED.

On the other hand, the parasitic emission (≈298 nm) is observed in the sample with the GSL-EBL, which is in agreement with the simulated emission result as shown in Fig. 7(a). The origin of this peak is considered an unintentional emission in the well of the GSL-EBL because the wavelength of the parasitic emission is nearly identical to the Al composition used for the well in the GSL-EBL. The detailed EL spectra of the DUV-LED with the GSL-EBL are indicated in Fig. 7(c). The EL intensity emitted at MQWs (280 nm) increases with current density from 20 mA to 80 mA. The emission ratio of the MQWs and well in the GSL-EBL is 2.03 at 20 mA; however, the emission ratio increases to 3.69 at current density of 80 mA, which means that the GSL-EBL is favorable at higher current densities. Fig. 7(d) shows the degree of optical power depending on the current injection for the DUV-LEDs with the S-EBL, G-EBL, and GSL-EBL. The intensities of the DUV-LEDs with the G-EBL and GSL-EBL are similar at 20 mA. However, the optical power of the GSL-EBL increases more drastically than that of the G-EBL with increasing current density. The DUV-LED fabrication will be conducted for further study on the chip level.

## Conclusions

We investigated the effect of modified EBLs on DUV-LEDs grown using HT-MOCVD. The TEM data showed the full-structure of the DUV-LED with MQWs and the EBL. The simulation data showed that the carrier injection to the MQWs was enhanced in the samples with the G-EBL and GSL-EBL. The polarization field in the GSL-EBL was particularly effective for multiplying hole concentration. The simulation showed that the injection efficiencies for the DUV-LEDs with the S-EBL, G-EBL, and GSL-EBL were 37%, 82%, and 88% through simulation, respectively. The EL results demonstrated a considerable improvement in the optical properties of the DUV-LED with the GSL-EBL. The optical power of this DUV-LED was approximately 17 times than that of the DUV-LED with the S-EBL at 80 mA.

## Conflicts of interest

There are no conflicts to declare.

## Supplementary Material
